# Is there a gap between artificial intelligence applications and priorities in health care and nursing management?

**DOI:** 10.1111/jonm.13851

**Published:** 2022-10-24

**Authors:** Yanjiao Chen, Paulo Moreira, Wei‐wei Liu, Michelle Monachino, Thi Le Ha Nguyen, Aihua Wang

**Affiliations:** ^1^ Research Center on Social Work and Social Governance in Henan Province Henan Normal University, Sociology Department Xinxiang China; ^2^ Shandong Provincial Qianfoshan Hospital Jinan Shandong China; ^3^ Departamento de Ciencias da Gestao (Gestao em Saude) Atlantica Instituto Universitario Oeiras Portugal; ^4^ School of Social Work Henan Normal University Xinxiang China; ^5^ Atlantica Instituto Universitario, Gestao em Saude Oeiras Portugal; ^6^ VNU University of Medicine and Pharmacy Vietnam National University Hanoi Vietnam; ^7^ Obstetrics Department Kunming Maternal and Child Hospital Kunming China

**Keywords:** nursing leadership, nursing management

## Abstract

**Aim:**

The article aims to outline a contrast between three priorities for nursing management proposed a decade ago and key features of the following 10 years of developments on artificial intelligence for health care and nursing management. This analysis intends to contribute to update the international debate on bridging the essence of health care and nursing management priorities and the focus of artificial intelligence developers.

**Background:**

Artificial intelligence research promises innovative approaches to supporting nurses' clinical decision‐making and to conduct tasks not related to patient interaction, including administrative activities and patient records. Yet, even though there has been an increase in international research and development of artificial intelligence applications for nursing care during the past 10 years, it is unclear to what extent the priorities of nursing management have been embedded in the devised artificial intelligence solutions.

**Evaluation:**

Starting from three priorities for nursing management identified in 2011 in a special issue of the Journal Nursing Management, we went on to identify recent evidence concerning 10 years of artificial intelligence applications developed to support health care management and nursing activities since then.

**Key Issue:**

The article discusses to what extent priorities in health care and nursing management may have to be revised while adopting artificial intelligence applications or, alternatively, to what extent the direction of artificial intelligence developments may need to be revised to contribute to long acknowledged priorities of nursing management.

**Conclusion:**

We have identified a conceptual gap between both sets of ideas and provide a discussion on the need to bridge that gap, while admitting that there may have been recent field developments still unreported in scientific literature.

**Implications for Nursing Management:**

Artificial intelligence developers and health care nursing managers need to be more engaged in coordinating the future development of artificial intelligence applications with a renewed set of nursing management priorities.

## 1 INTRODUCTION

Research on artificial intelligence promises innovative approaches to supporting health care management, clinical decision‐making and to conduct tasks not related to patient interaction, including nursing administrative activities and patient records. Yet, even though there has been an increase in international research and development of artificial intelligence applications for nursing care during the past 10 years, are these in tune with priorities identified for nursing management? How do nursing management priorities identified in 2011 relate to artificial intelligence applications for nursing developed till 2021? Has there been some level of miscommunication between artificial intelligence applications developers and nursing managers?

In other words, to what extent priorities in nursing management may have to be revised while adopting artificial intelligence or, alternatively, to what extent the direction of artificial intelligence development may need to be revised to contribute to acknowledged priorities of nursing management? Starting from the priorities for nursing management identified in 2011 in a special issue of the Journal Nursing Management (Parker & Hyrkas, 2011), we will go on to identify the most recent evidence concerning the trends on artificial intelligence applications focussing on nursing activities (Seibert et al., 2021). The final section of the article discusses identified gaps.

Additional evidence on nursing informatics (NI; Peltonen et al., 2016; Topaz et al., 2016) has identified the point of view of nursing specialists concerning what they consider and recommend to be key research and development trends and topics, including artificial intelligence, clinical decision support systems, big data research and care coordination. This evidence, published in between 2011 and 2021, corroborates the need to clarify the central question raised: Is there a gap between artificial intelligence applications development and priorities in nursing management?

The literature adopted for this article was identified adopting the guidance of a modified ‘argumentative literature review’ methodology as developed by McCullough et al. (2004) for evaluating topics while examining literature selectively in order to support or refute an argument. The purpose of this approach to literature review is to develop a body of literature that establishes a contrarian viewpoint. The authors identified the focussed questions of interest and conducted a literature search and review of pertinent articles on the topic of interest, collating and assessing the arguments based themes used by the relevant articles of interest and assessing the conclusions the authors used in supporting their arguments as explored in this article's subsequent sections.

## 2 NURSING MANAGEMENT PRIORITIES: AS PROPOSED IN 2011

Even though nursing management priorities have not been postulated in the format of a globally adopted protocol, for the purpose of updating the international debate, the article adopted the perspective of a leading publication in the field as guidance for one such clarification. Thus, the authors consider the perspective of the editors of the Journal of Nursing Management who, in 2011, posed the question, ‘where do we want nursing to be?’ In a special issue of the Journal, the international debate proposed was set on discussing the priorities for nursing management development (Parker & Hyrkas, 2011).

Three central themes were identified. The staff related management challenge of moving from satisfaction to retention, being one; developing practice and quality of care, being a second, and financial responsibility and sustainability, being a third. The three themes mirror a number of various previously published perspectives from which the authors proposed a set of key priorities, briefly clarified in the next paragraphs.

On what concerns the nursing management priority of evolving from satisfaction to retention, it stems from a general anticipation of nursing shortages and its consequences of which were by then considered potentially very negative for patients and health systems. In fact, in 2011, turnover rates of newly graduated RNs could be as high as 50–60%, which raised cost‐effectiveness issues considering the heavy recruitment and orientation resources applied to tackle this nursing management challenge and a worsening shortage of the nursing workforce. This problem has been identified for a long while (Armstrong‐Stassen & Cameron, 2003; Bergmann, 2006; Cohen, 1986) and has been receiving ongoing confirmation since 2011 (Dotson et al., 2013; Moreland et al., 2015; Polly et al., 2020).

Thus, some key management interventions to improve retention have been urged since, including reassessments of newly recruited nurses' workload, hours of work, adequacy of orientation to improve levels of organizational commitment, group cohesion and social support in hospital settings and time in contact with managers were also Identified as key nursing management interventions aimed at enhancing nurses retention (Parker & Hyrkas, 2011). The relevance of these recommendations has been demonstrated by several other authors since then (Antunes, 2022; Magbity et al., 2020; Norbye & Skaalvik, 2013).

On what concerns the nursing management priority of developing practice and quality of care, these have included best practices and evidence‐based practice (EBP) frameworks, infrastructure to support nursing research, staff familiarity to the available resources and protected time to provide guidance and direction for research efforts and knowledge transfer activities. These central factors identified in 2011 as fundamental required management improvements. Additionally, clinical supervision associated with lowering stress, improving practice development and the ethical issue of confidentiality in the context of whistle blowing, was also put forward in the context of this nursing management priority. Additionally, in 2011, it was also anticipated that inter‐cultural sensitivity and competencies would become relevant due to globalization and related increasing mobility of nurses and patients introducing different types of challenges to the nurse managers, directors and educators, and requiring everyone to re‐assess traditional roles, values, beliefs and practices. Other authors have, in the meantime, identified similar challenges (Antunes, 2022; Magbity et al., 2020; Sungur et al., 2019).

On what refers to the nursing management priority of financial responsibility and sustainability, the main challenges identified include budgetary and management control. Also relevant to clarify this priority was the perception that budgetary control activities and role varied across health care managers depending on their professional background/education and gender. This generated the assumption that there should be specific educational requirements on economics for a manager in a position that holds major financial responsibilities. Hence, the concept of Accountable Carei was added. This being defined as the process of assuming shared responsibility for the quality and cost of care provided to with a focus on health care value, that is to say care that is effective, involves patients in decision making and reduces delivery of care not needed. Other authors have, in the meantime, contributed to update these challenges (Antunes, 2022; Norbye & Skaalvik, 2013; O'Donnell et al., 2012; Townsend et al., 2012).

In this manner, we put forward a fundamental question to be tackled in our next section. In spite of all the evidence available, have artificial intelligence applications considered these nursing management challenges in their applied developments?

## 3 ARTIFICIAL INTELLIGENCE PRIORITIES FOR NURSING MANAGEMENT: THE STATUS IN 2021

In this section, we explore available recent evidence to understand and discuss how artificial intelligence applications have been evolving overall and to what extent we can corroborate that these have been developed within the nursing management priorities identified in the previous section.

Do artificial intelligence applications have room for improvement on their understanding of nursing management priorities?

Although consensus on the potential of health technologies powered by artificial intelligence to enhance nursing practice has been reported (Buchanan et al., 2020), a more recent study (Seibert et al., 2021) has shed light upon the circumstantial shortcoming s in application scenarios for artificial intelligence in nursing care settings. These are identified in the literature during the period of 10 years starting from 2011.

The definition of artificial intelligence adopted in this article is that it consists of algorithms that enable learning from data sets to achieve intelligent, goal‐oriented action. In other words, algorithms that should be developed to serve the nursing management priorities.

Artificial intelligence developers are understood as the organizations (of both commercial or non‐commercial nature) and related teams of programmers developing artificial intelligence solutions made available to health systems around the world.

First, it comes as a fact that in the published literature, hospitals were the most prominent setting of artificial intelligence applications in the previous 10 years of development, followed by independent living at home. In contrast, fewer applications were identified for nursing homes, home care and ambulatory long‐term care.

In fact, the context of direct nursing care artificial intelligence is reported in the literature to organize care processes and support care‐dependent people or family caregivers through tracking, monitoring or classifying activities. Health‐related data are additionally supported by applications for care coordination including nurse rostering and scheduling. Detecting, classifying and preventing falls, as well as recognizing, classifying and reducing alarms, and predicting and classifying pressure ulcers were further purposes of introducing artificial intelligence to nursing care (Seibert et al., 2021).

However, to the probable surprise of nursing and health care managers today, few studies have reported an emphasis of artificial intelligence applications on clinical or organizational outcomes. This apparent predicament is further aggravated by the fact that artificial intelligence applications are short in data gathered outside laboratory conditions. Also, the universally accepted classification of artificial intelligence subfields relevant to health, which could act as a point in favour of enhancing artificial intelligence in nursing practice, was reported to be missing. These findings had already been exposed in other previous recent studies (Buchanan et al., 2020; Kikuchi, 2020; Wahl et al., 2018).

Hence, since 2011, Machine Learning (ML) algorithms were by far the dominant development approach in detriment of expert or hybrid systems with rule‐based expert systems only adding to 11.6% of the studies published since 2011 (Seibert et al., 2021).

It is also evident that artificial intelligence application developments for nursing have been focusing on image and signal processing with tracking, monitoring of patients and consumables, especially in the hospital context, and much less relevant to other settings of care (i.e., primary care and home care), even though this approach contributes to organize and prioritize activities that can be applied to boost the efficiency of nursing care procedures. In contrast, studies have focused less often on the processing of human language (Natural Language Processing or NLP) as also recently further explored (Jacennik et al., 2022).

In addition to technical or computational requirements, further requirements concerning the specific context of nursing care are scarce and mainly tackle overarching topics, such as data privacy, safety and acceptance. The same holds for Ethical, Legal and Societal Issues (ELSIs), which, for instance, have not been reflected or discussed in the majority of studies using real‐world scenarios (Seibert et al., 2021).

Further to these predicaments, as demonstrated by the central systematic review under scrutiny, the health, wellbeing and satisfaction of the caregiver were addressed in a very small number of cases. Published research in real‐world settings has also not given any prominent role to the support of care‐dependent people and patient education and no research conducted in a real‐world setting focused on the health of caregivers or nurses. Additionally to these findings, only a few publications went beyond proof‐of‐concept studies or laboratory experiments and applied artificial intelligence in real‐world scenarios, and even fewer studies have assessed the effects of artificial intelligence on clinical and organizational outcomes (Seibert et al., 2021). The particularly important issue is further corroborated by other studies that suggest that there are certain gaps in artificial intelligence applications used in outcomes research across therapeutic areas and further considerations will be needed before artificial intelligence usage can be incorporated into health technology assessment decision‐making processes (Bélisle‐Pipon et al., 2021).

Hence, other recent systematic and scoping reviews on the application of artificial intelligence in nursing research (as well as in practice and emerging trends), covering original research published, suggest that the focus has been on ML methods, such as deep learning, or on health technologies that incorporate artificial intelligence approaches themselves, such as robots or clinical decision support systems. Various application scenarios have been identified, including clinical or organizational outcomes (especially patient falls), admission decisions in emergency medicine, high‐definition image recognition and socially assistive robots or health care assistant chatbots. There has been an increase in research and research needs discussion highlighting possibilities for the future development of artificial intelligence in nursing care. However, the importance of collaborative, interdisciplinary research has been generally underscored (Ampavathi & Vijaya Saradhi, 2022; Apell & Eriksson, 2021; Jacennik, 2022).

## 4 WHAT ARE THE GAPS?

In the previous sections, we have presented simplified sets of principles influencing two key areas of health systems development. On the one hand, we clarified the key nursing management priorities to tackle key health care management challenges as assumed in 2011 in an internationally relevant scientific publication.

On the other hand, we have presented an overview of the focus of artificial intelligence developers when it comes to emanating solutions for nursing management. There seems to be a gap when we set their prime concerns side by side.

In fact, as presented in Table 1, nursing management priorities proposed in 2011 focussed on three key ideas. One, focussing on human resources management, would be evolving from staff satisfaction to retention through revising nurses' workload, balancing hours of work and adequacy of orientation from leaders, while enhancing group cohesion and social support. Another second priority was that of developing the practice and quality of nursing care, through the support and access to sources of evidence‐based‐practice (EBP), support advanced nursing research. Also, adapt research resources for those purposes via more efficient knowledge transfer to improve clinical supervision and whistle blowing, adding to these the improvement of intercultural competencies to work with international patients and international staff. Additionally, a third priority was the expectation of the advancement of competencies on financial responsibility practiced by nurses towards sustainability, by improving nurses' capacities on budgetary and management control while developing specific educational support on economics; accountable care; health care value and accountable care.

**TABLE 1:** Contrasting nursing management priorities and artificial intelligence applications prime concerns

Three nursing management priorities in 2011 and some key related activities	Artificial intelligence applications as developed between 2011 and 2021
**From staff satisfaction to retention**: nurses' workload, hours of work and adequacy of orientation, group cohesion and social support **Developing practice and quality of nursing care**: Evidence‐based‐practice, nursing research, resources, knowledge transfer, clinical supervision and whistle blowing, intercultural competencies; **Financial responsibility and sustainability**: Budgetary and management control, specific educational support on economics; accountable care; health care value; accountable care:	Main health system focus: Hospitals and independent living at home Organizational managerial contributions: Patient tracking and monitoring, classification of activity, care coordination and communication Technological focus: Image and signal processing, machine learning systems Patient care contributions: Patient fall detection, and predicting and classifying pressure ulcers

To what extent can we argue that artificial intelligence applications have taken in consideration any of the nursing management priorities proposed in 2011?

An answer to this question can be suggested from evidence published in 2021. As presented in Table 1, we can now have an overview of the main areas of development devised by artificial intelligence developers.

On what concerns key contributions with a health system perspective, the focus has been on hospitals and independent living at home. On what concerns managerial contributions of artificial intelligence applications to health care organizations, evidence suggests a focus on patient tracking and monitoring, classification of activity and broad care coordination. The additional theme of technological developments brought about contributions on image and signal processing and ML systems. When we consider contributions of artificial intelligence to patient care, evidence suggests a focus on patient fall detection as well as predicting and classifying pressure ulcers (on a rare emphasis on specific nursing care issues).

From this summary of evidence, put in contrast between 2011 and in 2021, we can therefore argue that we have identified a gap between the proposed priorities for the improvement of nursing management and the actual developments of artificial intelligence applications positioned as solutions for health care.

In fact, following the evidence explored in the previous sections, we can raise some questions for the international debate. Where are the artificial intelligence applications to support the evolution from staff satisfaction to staff retention? Namely, artificial intelligence solutions that contribute to revising nurses' workload, balance hours of work aimed at enhancing group cohesion and social support.

Also, evidence from 2021 is not clear on how has artificial intelligence contributed to developing the practice and quality of nursing care, through the support and access to sources of evidence‐based‐practice or more efficient knowledge transfer to improve clinical supervision or even the improvement of intercultural competencies.

Additionally, the same evidence is not clear on how have artificial intelligence applications contributed to the advancement of competencies on financial budgetary and management control while developing specific educational support on economics; accountable care; health care value and accountable care.

We do not deny the value and interest of artificial intelligence applications for nursing care. And yet we have identified a set of gaps that needs to be discussed internationally.

Further to the gaps for practice identified, a further critical gap identified has also been clarified and it concerns research on the limitations of the clinical storage systems, noise removal methods and multi‐disease prediction models (Ampavathi & Vijaya Saradhi, 2022). As these are related to innovation system performance, gaps are primarily restricted by the system weaknesses of limited resources and insufficient communication from leading health care professionals regarding their needs for improving health care using artificial intelligence technology innovations. In other words, evidence suggests that to improve innovation system performance, policy interventions intended to increase available resources and to formulate common vision and mission statements to improve health care with artificial intelligence technology innovations must be encouraged. This supports our view of an existing gap between nursing management priorities and artificial intelligence developers' perspectives (Apell & Eriksson, 2021). Other recent evidence on NI research has also identified the need to focus in key research topics including clinical decision support systems, big data research for nursing management and patient care coordination. The findings show that top research trends have changed when compared to findings in 2015 and that NI research is evolving rapidly. This puts pressure on NI education at all levels, namely to continue and advance these topics in nursing education curriculums and clinical practice and ensuring nurses are knowledgeable about the technologies and their use (Peltonen et al., 2016; Topaz et al., 2016).

## 5 IMPLICATIONS FOR NURSING MANAGEMENT

The previous sections of this article sustain the clear conclusion that there must be a more deliberate coordination between current nursing management priorities and the development and deployment of artificial intelligence to address the defined priorities, as already proposed in 2011. To address this issue in a clear and actionable manner, we summarize in this section a set of actionable items for nursing managers.

Overall, there seems to be have been an apparent distance between the definition of nursing management priorities and the development of artificial intelligence applications between 2011 and 2021. It is necessary, therefore, to focus the international debate on bridging gaps between nursing management priorities and artificial intelligence developers' priorities. Nursing leaders must make additional efforts to integrate and lead artificial intelligence development groups at health system level as well as at top organizational levels. On what concerns the essential contents of artificial intelligence, one key implication of our analysis is that a better understanding of quality issues related to the specific activities of nursing care is required to be adopted by artificial intelligence developers.

Another concern is the need to add a contribution of artificial intelligence to nursing staff management. Moving from satisfaction to retention in staff management is a priority of nursing management since 2011.

An additional actionable item for nursing managers is to integrate into artificial intelligence solutions functions for managing the quality of care. Also, functions to support financial responsibility and sustainability, being also to be expected.

A gap has been identified on what concerns the few applications developed for nursing homes, home care and ambulatory long‐term care, as well as few relevant artificial intelligence solutions for other settings of care beyond hospitals, namely: primary care and home care as these contribute to organize and prioritize activities to boost the efficiency of nursing care procedures.

One other implication for nursing management and an actionable item for nursing managers is the development of artificial intelligence solutions to support care‐dependent people and patient education programs.

There are three key additional challenges for nursing management generated by our analysis, namely, pertaining to actionable items for management. One is the need to improve nursing leadership competencies in the innovation process of developing artificial intelligence solutions focussing on the real needs of nursing care. A second actionable action for management is the need to improve the contribution of artificial intelligence to health care quality monitoring in and beyond hospitals in an integrated care perspective as widely advocated by Health Technology Assessment guidelines. A third implications is that it has in a whole decade of developing solutions of artificial intelligence applied to nursing management, the key perspectives of health care quality management have not been fully integrated into the solutions identified, and this should be a priority for nurse managers in the process of influencing the development of artificial intelligence solutions. Hence, the challenge of adding artificial intelligence into nursing education curricula and research agenda remains as a crucial development (Moreira, 2022; Peltonen et al., 2016).

Yet, a word of caution the above implications. There may be recent field developments that are not yet available through published scientific literature.

## 6 CONCLUSIONS

Artificial Intelligence remains an exciting development in health care management. As an innovative contribution for health systems development, the process will need ongoing improvements in its implementation and change management practice. There should be more deliberate coordination between nursing management priorities and the development and deployment of artificial intelligence to address those priorities. We have addressed this question in a clear and actionable manner. However, how can these gaps be addressed realistically? This is a key challenge for the coming years. Establishing operational commissions for nursing management and artificial intelligence at international or national levels would be a fundamental expectation resulting from the awareness of the gaps identified. These commissions should integrate senior nursing managers leading the process of influencing the setting of functions offered by artificial intelligence solutions, rather than the process of simply adopting already existing artificial intelligence functions to health care.

Interestingly, the recent report on ‘The Future of Nursing 2020‐2030’ (National Academies of Sciences, Engineering, and Medicine, 2021), although focussing on the specific USA context, does not put an emphasis on the role of nurses on health systems innovations associated to health care research trends (Lloyd Williams, 2022) or to artificial intelligence. It rather proposes the investment in the goals of achieving health equity, removing barriers to expand the scope of practice, establish better measurements of the value of services provided by RNs, developing nurses' ability to address social determinants of health and the needs of the community, including in response to public health emergencies and disasters. It also recommends that research projects are used to demonstrate the racial and ethnic diversity within the newer classes of nurses this being presented as an important step towards achieving health equity. Additional recommendations of this report include a managerial focus on tackling poverty and other health inequities through simulation‐based education, and new guidelines for evidence‐based quality indicators for nursing education programs to support a broader understanding of quality nursing education. A summarized interpretation of this report could be that it brings forth new nursing management priorities set in 2021, which are likely to influence health systems and policy‐making around the world.

In this manner, we can propose a closing discussion point. Admitting that during the past decade, artificial intelligence solutions seem to have ignored key nursing management priorities as proposed in 2011 (Parker & Hyrkas, 2011), to what extent the new priorities proposed in the May 2021 report (National Academies of Sciences, Engineering, and Medicine., 2021) and argeued by other authors (Graili, et al., 2021; McGrow, 2019), will again be ignored by upcoming artificial intelligence developments?

## CONFLICT OF INTEREST

The authors declare that there is no conflict of interest.

## ETHICS STATEMENT

Our study did not require an ethical board approval because the article is a commentary and does not use any patient data.

## Data Availability

Data sharing not applicable to this article as no datasets were generated or analysed during the current study.
